# Mesenchymal Stem Cell-Mediated Functional Tooth Regeneration in Swine

**DOI:** 10.1371/journal.pone.0000079

**Published:** 2006-12-20

**Authors:** Wataru Sonoyama, Yi Liu, Dianji Fang, Takayoshi Yamaza, Byoung-Moo Seo, Chunmei Zhang, He Liu, Stan Gronthos, Cun-Yu Wang, Songtao Shi, Songlin Wang

**Affiliations:** 1 Center for Craniofacial Molecular Biology, University of Southern California School of Dentistry, Los Angeles, California United States of America; 2 Salivary Gland Disease Center and the Molecular Laboratory for Gene Therapy, Capital Medical University School of Stomatology Beijing, China; 3 Department of Oral and Maxillofacial Rehabilitation, Okayama University Graduate School of Medicine, Dentistry and Pharmaceutical Sciences Okayama, Japan; 4 Department of Oral and Maxillofacial Surgery, School of Dentistry, Dental Research Institute, Seoul National University Seoul, Korea; 5 Peking University School of Stomatology Beijing, China; 6 Mesenchymal Stem Cell Group, Division of Haematology, Institute of Medical and Veterinary Science Adelaide, South Australia, Australia; 7 Laboratory of Molecular Signaling and Apoptosis, Department of Biologic and Materials Sciences and Program in Cellular and Molecular Biology, University of Michigan Ann Arbor, Michigan, United States of America; Emory University, United States of America

## Abstract

Mesenchymal stem cell-mediated tissue regeneration is a promising approach for regenerative medicine for a wide range of applications. Here we report a new population of stem cells isolated from the root apical papilla of human teeth (SCAP, stem cells from apical papilla). Using a minipig model, we transplanted both human SCAP and periodontal ligament stem cells (PDLSCs) to generate a root/periodontal complex capable of supporting a porcelain crown, resulting in normal tooth function. This work integrates a stem cell-mediated tissue regeneration strategy, engineered materials for structure, and current dental crown technologies. This hybridized tissue engineering approach led to recovery of tooth strength and appearance.

## Introduction

Regeneration of a functional and living tooth is one of the most promising therapeutic strategies for the replacement of a diseased or damaged tooth [1]–[3]. Recent advances in dental stem cell biotechnology and cell-mediated murine tooth regeneration have encouraged researchers to explore the potential for regenerating living teeth with appropriate functional properties [4]–[6]. Murine teeth can be regenerated using many different stem cells to collaboratively form dental structures *in vivo* [4], [5], [7]. In addition, dentin/pulp tissue and cementum/periodontal complex have been regenerated by human dental pulp stem cells (DPSCs) and periodontal ligament stem cells (PDLSCs), respectively, when transplanted into immunocompromised mice [8], [9]. However, owing to the complexity of human tooth growth and development, the regeneration of a whole tooth structure including enamel, dentin/pulp complex, and periodontal tissues as a functional entity in humans not possible given available regenerative biotechnologies.

The spatially and temporally organized microenvironment of the tooth bud and its surrounding tissues permits growth and development of the crown and roots, resulting in formation and eruption of the tooth [10]. Root development involves dentin formation, cementum generation, instruction of epithelium, and tooth eruption. From a clinical perspective, the most important part of the tooth is the root which supports for a (natural or artificial) crown. The crown alone cannot fulfill normal tooth function without a viable root. In contrast, the wide use of synthetic crowns to replace a damaged natural crowns has been widely applied in dental clinics with excellent therapeutic outcomes [11].

Although dental implant therapies have achieved long-term success in the clinic for the recovery of tooth function, the dental implants require pre-existing high-quality bone structures for supporting the implants [12], [13]. Reconstruction of teeth in patients without adequate bone support would be a major advance. Stem cell-mediated root regeneration offers opportunities to regenerate a bio-root and its associated periodontal tissues, which are necessary for maintaining the physiological function of teeth. The purpose of this study is to explore the potential for reconstructing a functional tooth in miniature pigs (minipigs), in which a bio-root periodontal complex is built up by postnatal stem cells including stem cells from root apical papilla (SCAP) and PDLSCs, to which an artificial porcelain crown is affixed. This hybrid strategy of autologous dental stem cell engineering may be applicable to human tooth regeneration. Furthermore, functional tooth restoration in swine may shed light on human tooth regeneration in the future because of the close similarities between swine and human dental tissues [14], [15].

## Results

### Isolation and transplantation of SCAP

**Figure 1:**
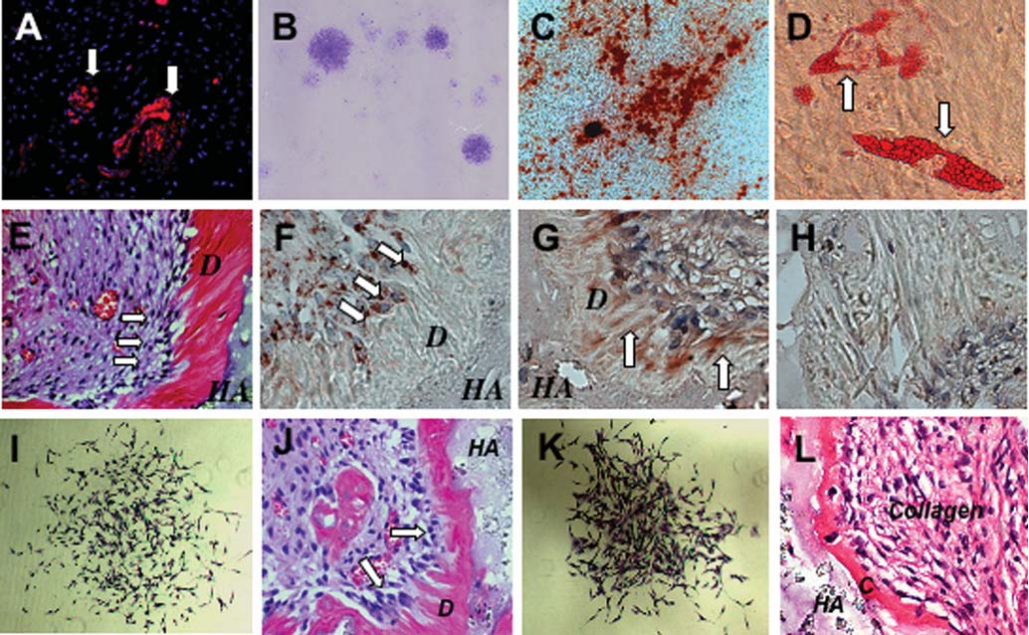
Isolation of Stem Cells from Root Apical Papilla (SCAP). (**A**) Human apical papilla tissue was positive for STRO-1, an early mesenchymal progenitor marker, staining by immunofluorescence (arrows). (**B)** Single colonies were formed after human SCAP were plated at a low density (5×10^3^/T25 flask) and cultured for 10 days. (**C**) When human SCAP were cultured in odontogenic/osteogenic inductive conditions containing L-ascorbate-2-phosphate, dexamethasone, and inorganic phosphate for 4 weeks, mineralized nodules were found by Alizarin red S staining. (**D**) Cultured human SCAP formed Oil red O positive lipid clusters following 5 weeks of adipogenic induction in the presence of 0.5 mM isobutylmethylxanthine, 0.5 µM hydrocortisone, 60 µM indomethacin, and 10 µg/ml insulin. (**E**) Eight weeks after transplantation in immunocompromised mice, human SCAP differentiated into odontoblasts (arrows) that formed dentin (*D*) on the surfaces of a hydroxyapatite tricalcium (*HA*) carrier. (**F**) Immunohistochemical staining showed that human SCAP differentiated into odontoblasts (arrows) that were positive for anti-human specific mitochondria antibody staining. (**G**) Immunohistochemical staining showed that human SCAP-generated dentin (*D*) was positive for anti-DSP antibody staining (arrows). (**H**) Pre-immunoserum negative control of human SCAP transplant. (**I**) SCAP isolated from swine were capable of forming a single colony cluster when plated at a low cell density. (**J**) When transplanted into immunocompromised mice for 8 weeks, swine SCAP differentiate into odontoblasts (arrows) to regenerate dentin (*D*) on the surface of the hydroxyapatite carrier (*HA*). (**K**) Swine PDLSCs were capable of forming a single colony cluster. (**L**) After transplantation into immunocompromised mice, swine PDLSCs formed cementum (*C*) on the surface of hydroxyapatite carrier (*HA*). Collagen fibers were found to connect to newly formed cementum.

The mechanism of the contribution of stem progenitors to root formation remains to be elucidated. Here, we found that human root apical papilla tissue on the exterior of the root foramen area demonstrated positive staining for mesenchymal stem cell surface molecule STRO-1 (Figure 1A). The root apical papilla might contain a population of stem/progenitor cells. To identify putative stem cells, single-cell suspensions were generated from human root apical papillae collected from extracted third molars of 18–20 years old adult volunteers, following collagenase/dispase digestion. When cultured at a low cell density, they formed adherent clonogenic cell clusters (CFU-F, colony forming unit, fibroblastic) (Figure 1B), similar to those observed for various mesenchymal stem cell populations. To investigate the potential of SCAP to undergo odontoblastic/osteoblastic differentiation, multiple colony-derived SCAP at passage two were supplemented with L-ascorbate-2-phosphate, dexamethasone, and inorganic phosphate to induce mineralization *in vitro* as described previously [8], [9]. Small round Alizarin Red-positive nodules formed in the SCAP cultures after four weeks of induction, indicating calcium accumulation *in vitro* (Figure 1C). Moreover, cultured SCAP were capable of differentiating into other cell types such as adipocytes (Figure 1D), analogous to DPSCs and bone marrow mesenchymal stem cells (BMMSCs) [8].

To validate the capacity of SCAP to differentiate into functional dentinogenic cells, *ex vivo* expanded-SCAP were transplanted into immunocompromised mice, with hydroxyapatite/tricalcium phosphate (HA/TCP) as a carrier. A typical dentin structure was regenerated, in which a layer of dentin tissue formed on the surface of the HA/TCP along with connective tissue (Figure 1E). The newly formed dentin was positive for anti-DSP antibody staining, and dentin-forming cells stained with anti-human-specific mitochondria antibody (Figure 1F–H), suggesting that the donor derived human SCAP had formed the dentin *in vivo*. In order to prepare for autologous dental stem cells for functional tooth regeneration in swine, we isolated single colony-derived swine SCAP and PDLSCs and transplanted them into immunocompromised mice to regenerate dentin and cementum, respectively (Figure 1I–1L), equivalent to that of human SCAP and PDLSCs.

**Figure 2:**
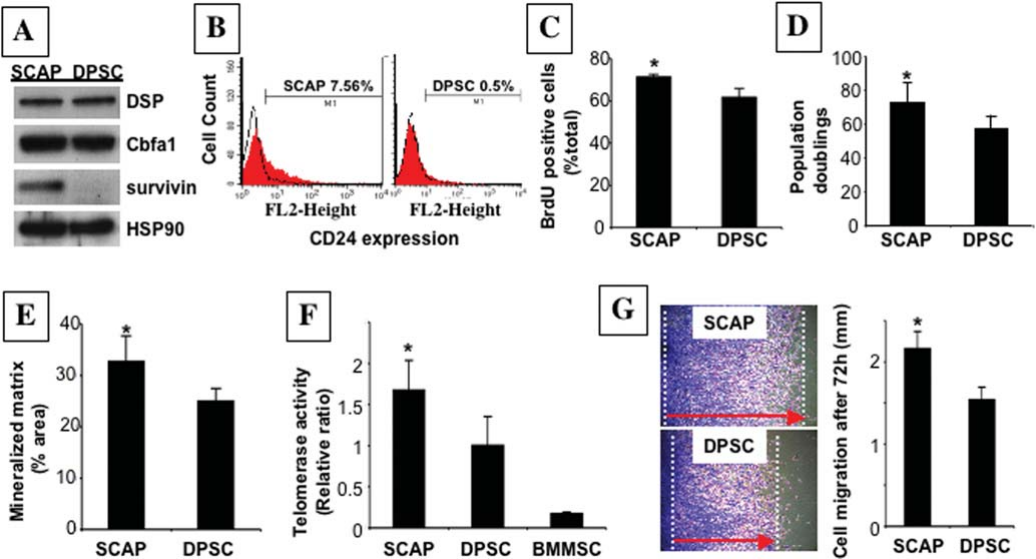
Characterization of human SCAP in comparison with DPSCs. (**A**) Western blot analysis to confirm protein expression of genes identified in microarray studies showed greater abundance of survivin in SCAP than in DPSCs, with similar levels of DSP and Cbfa1/Runx2. (**B**) Flow cytometric analysis showed that *ex vivo* expanded SCAP contained approximately 7.5% CD24-positive cells, but DPSCs exhibited 0.5% positive staining for CD 24. (**C**) The proliferation rates of SCAP and DPSCs, derived from the same tooth, were assessed by co-culture with BrdU for 6 hours. The number of BrdU-positive cells was presented as a percentage of the total number of cells counted from five replicate cultures. SCAP showed a significantly higher proliferation rate in comparison with DPSCs (* *P* = 0.0042). (**D**) Single colony-derived SCAP were able to proliferate to over 70 population doublings, which was significantly higher than DPSCs (* *P* = 0.0192). (**E**) Dentin regeneration capacity of SCAP was significantly higher than that of DPSCs when transplanted into the same immunocompromised mice (* *P* = 0.0489) using Scion Image analysis system (Scion Image, Rockville, MD). (**F**) SCAP showed a significant higher telomerase activity than DPSCs at passage 1 (* *P* = 0.015). Cultured BMMSCs at passage 1 were used as a negative control to show an absence of telomerase activity. The telomerase activity was assessed by real time PCR based quantitative telomerase detection kit as described in Materials and Methods. (**G**) Cell motility assessed by a scratch assay. A representative area of SCAP and DPSCs at 72 hours after scratch was presented. Red arrows indicate the ranges of cell migration during 72 hours (* *P* = 0.0033).

**Figure 3:**
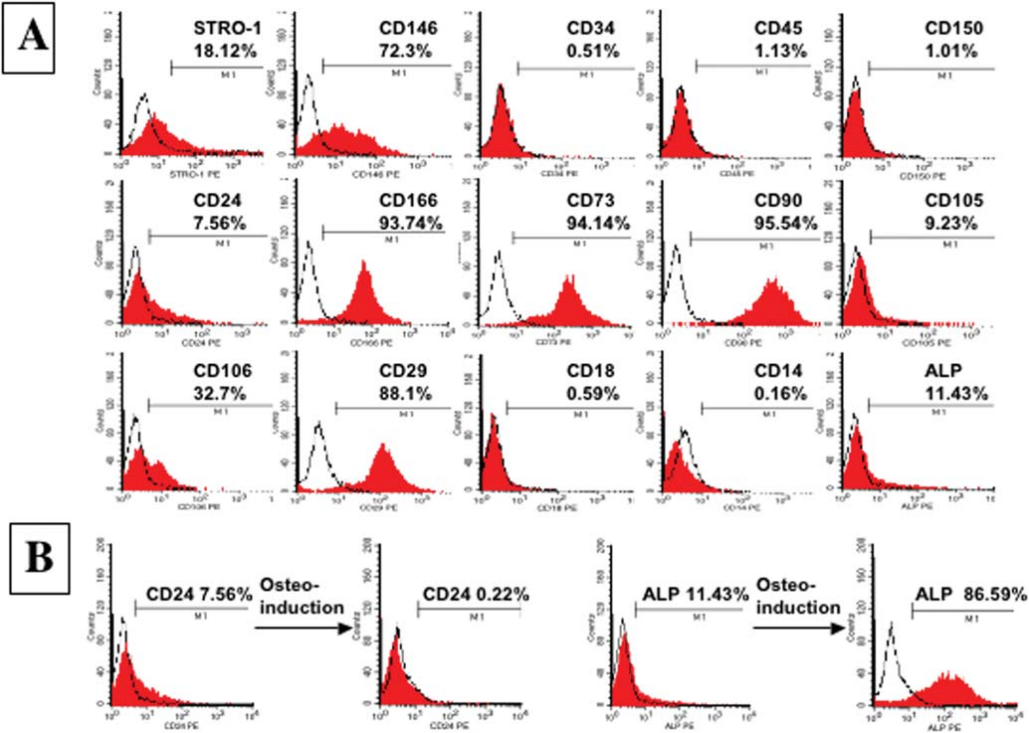
Surface Molecule Characterization of human SCAP. (**A**) Flow cytometric analysis of cultured SCAP at passage 1 revealed expression of STRO-1 (18.12%), CD146 (72.3%), CD24 (7.56%), CD166 (93.74%), CD73 (94.14%), CD90 (95.54%), CD105 (9.23%), CD106 (32.7%), CD29 (88.1%) and ALP (11.43%), but was negative for surface molecules CD18, CD14, CD34, CD45, and CD 150. (**B**) After 2 weeks osteo-induction *in vitro* with L-ascorbate-2-phosphate, dexamethasone, and inorganic phosphate, SCAP differentiated into odontoblasts with a decrease in CD24 expression from 7.56% to 0.22%. In contrast, ALP expression increased significantly from 11.43% to 86.59%.

We next addressed whether SCAP and DSPCs were the same or distinct mesenchymal stem cell populations, because both expressed osteo/odontogenic markers and generate mineralized tissue when transplanted into immunocompromised mice. First, we collected SCAP and DPSCs from the same human tooth and grew them under exactly same conditions. According to a cDNA microarray profile comparison (data not shown), there are many genes differentially expressed between these two stem cell populations. Among these genes, we selected survivin as one example, and confirmed its higher expression in SCAP by western blot (Figure 2A). We also identified that CD 24 as a specific surface marker of SCAP by flow cytometric analysis (Figure 2B). During odontogenic differentiation *in vitro*, SCAP lost the expression of CD24 with an up-regulated expression of alkaline phosphatase (ALP) (Figure 3B). In addition, SCAP showed a significantly higher rate of bromodeoxyuridine (BrdU) uptake (Figure 2C), and increased number of population doublings (Figure 2D). The SCAP population also demonstrated an elevated tissue regeneration capacity (Figure 2E), higher telomerase activity than that of DPSCs from the same tooth (Figure 2F), and an improved migration capacity in a scratch assay (Figure 2G), when compared to DPSCs from the same tooth. Collectively, these studies suggested that SCAP are a unique population of postnatal stem cells distinct from DPSC.

### Surface molecule characterization of SCAP

To characterize SCAP by surface molecules, we used flow cytometric analysis to demonstrate that SCAP at passage 1 expressed many surface markers including STRO-1, ALP, CD24, CD29, CD73, CD90, CD105, CD106, CD146, CD166 and ALP but were negative for CD34, CD45, CD18 and CD150 (Figure 3A). STRO-1 and CD146 have been identified as early mesenchymal stem cell markers present on both BMMSCs and DPSCs [8], [16]. Here we found that CD24 appears to be a specific marker for SCAP, not detectable in other mesenchymal stem cells including DPSCs and BMMSCs (data not shown). In response to osteogenic induction conditions in culture, SCAP begin to down regulate their expression of CD24 while gaining expression of ALP (Figure 3B). Our experimental evidence suggests that SCAP derived from a developing tissue may represent a population of early progenitors that have advantages for use in tissue regeneration.

### Functional tooth regeneration

**Figure 4:**
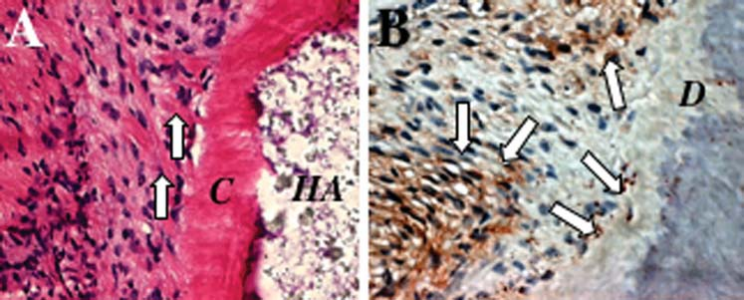
Comined human SCAP/PDLSC-mediated tissue regeneration. (**A**) On the outside of the HA/TCP carrier (*HA*), PDLSCs can form structures resembling Sharpey's fibers (arrows) connecting with newly formed cementum (*C*) on the surface of HA/TCP particles (*HA*). (**B**) Immunohistochemical staining showed that SCAP/PDLSC-generated tissues were positive for human specific mitochondria antibody staining (arrows).

Identification of SCAP provides an opportunity to pursue root regeneration using this high-quality “young” postnatal stem cell derived from 18–20 years old adult vounteers. To play a functional role *in vivo*, the root has to connect with the periodontal ligament to ensure correct positional stability and support *in situ*. Therefore, we used both human SCAP and PDLSCs to generate dentin and PDL on a HA/TCP carrier, in order to mimic a bio-physiological root/periodontal set-up *in vivo*. Eight weeks after transplantation, the human PDLSCs were able to form cementum on the surface of HA/TCP carrier and Sharpey's fibers, characterized histologically to have collagen fibers anchored into the cementum (Figure 4 A, B). These data suggested that use of combined mesenchymal stem cell populations provide a basis for root/periodontal tissue regeneration.

**Figure 5:**
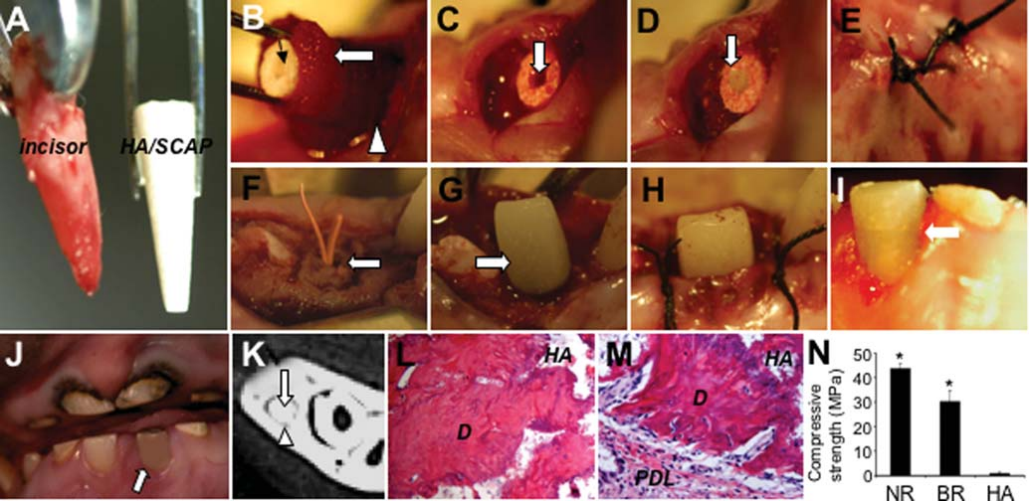
Swine SCAP/PDLSC-mediated root/periodontal structure as an artificial crown support for the restoration of tooth function in swine. (**A**) Extracted minipig lower incisor and root-shaped HA/TCP carrier loaded with SCAP. (**B**) Gelfoam containing 10×10^6^ PDLSCs (open arrow) was used to cover the HA/SCAP (black arrow) and implanted into the lower incisor socket (open triangle). (**C**) HA/SCAP-Gelfoam/PDLSCs were implanted into a newly extracted incisor socket. A post channel was pre-created inside the root shape HA carrier (arrow). (**D**) The post channel was sealed with a temporary filling for affixing a porcelain crown in the next step. (**E**) The HA/SCAP-Gelfoam/PDLSC implant was sutured for 3 months. (**F**) The HA/SCAP-Gelfoam/PDLSC implant (arrow) was re-exposed and the temporary filling was removed to expose the post channel. (**G**) A pre-made porcelain crown was cemented to the HA/SCAP-Gelfoam/PDLSC structure. (**H**) The exposed section was sutured. (**I** and **J**) Four weeks after fixation, the porcelain crown was retained in the swine after normal tooth use as shown by open arrows. (**K**) After 3 months implantation, the HA/SCAP-Gelfoam/PDLSC implant had formed a hard root structure (open arrows) in the mandibular incisor area as shown by CT scan image. A clear PDL space was found between the implant and surrounding bony tissue (triangle arrows). (**L** and **M**) H&E staining showed that implanted HA/SCAP-Gelfoam/PDLSC contains newly regenerated dentin (*D*) inside the implant (**L**) and PDL tissue (*PDL*) on the outside of the implant (**M**). (**N**) Compressive strength measurement showed that newly formed bio-roots have much higher compressive strength than original HA/TCP carrier (* *P* = 0.0002), but lower than that in natural swine root dentin (* *P* = 0.003) (NR: natural minipig root, BR: newly formed bio-root, HA: original HA carrier).

To accomplish functional tooth regeneration, we used swine because of the similarities in swine and human orofacial tissue organization. Swine SCAP were loaded into a root-shaped HA/TCP block that contained an inner post channel space to allow the subsequent installation of a porcelain crown (Figure 5A). A lower incisor was extracted and the extraction socket was further cleaned with a surgical bur to remove remaining periodontal tissues (Figure 5A). The HA/TCP block containing SCAP was coated with Gelfoam (Pharmacia Canada Inc., Ontario, Canada) containing PDLSCs and inserted into the socket and sutured for 3 months (Figure 5B–E). CT examination revealed a HA/SCAP-Gelfoam/PDLSC structure growing inside the socket with mineralized root-like tissue formation and periodontal ligament space. The surface of the implanted HA/SCAP-Gelfoam/PDLSC structure was surgically re-opened at three months post-implantation, and a pre-fabricated porcelain crown resembling a minipig incisor was inserted and cemented into the pre-formed post channel inside the HA/TCP block (Figure 5F–H). After suture of the surgical opening, the porcelain crown was retained in situ and subjected to the process of tooth function for four weeks (Figure 5I, J). CT and histologic analysis confirmed that the root/periodontal structure had regenerated (Figure 5K–M). Moreover, newly formed bio-roots demonstrated a significantly improved compressive strength than that of original HA/TCP carriers after six-month implantation (Figure 5N). These findings suggest the feasibility of using a combination of autologous SCAP/PDLSCs in conjunction with artificial dental crowns for functional tooth regeneration.

## Discussion

It is generally believed that the dental papilla contributes to tooth formation and eventually converts to pulp tissue within the pulp chamber. In this study, we found that the root apical papilla contained mesenchymal stem cells that appear to have a greater capacity for dentin regeneration than DPSCs. These findings suggest that developing tissue may contain a good stem cell resource for tissue regeneration. SCAP represent a novel population of multipotent stem cells as demonstrated by their capacity to develop into odontoblast-like cells and adipocytes *in vitro*. This cell population was found to express high levels of survivin and telomerase, which are both important molecules in mediating cell proliferation. In addition, CD24, marker for undifferentiated SCAP, which is downregulated following odontogenic differentiation. These data support the notion that SCAP are a unique population of postnatal stem cells.

Although dental pulp contains DPSCs with dentin/pulp regenerative capacity, developing tissue-derived SCAP showed a superior tissue regeneration potential than that of DPSCs. SCAP collected from just one tooth are capable of providing a large number of stem cells probably sufficient for human transplantation because they have high proliferative potential, reflected in high telomerase activity [17]. Human SCAP-mediated tissue regeneration may offer a promising cell-based therapy for root regeneration.

Since most human tissue at the developing stage is not clinically available for stem cell isolation, this apical papilla stem cell population, accessible in dental clinical practice, is unusual. SCAP can be isolated from extracted wisdom teeth. Therefore, it may be possible to bank these high quality dental stem cells for future autologous use. Their survival in freeze-thawing requires exploration. Allogeneic cells may be another resource of stem cells for treating those aged individuals who have already have had their wisdom teeth extracted, however the immunogenicity of these cells requires further study.

Although newly formed bio-roots show a lower compressive strength than that of natural swine root dentin, they seemed capable of supporting porcelain crown and resulted in normal functions. It may be possible to improve the compressive strength and hardness of the bio-roots by selecting optimal bioengineered materials and by optimizing the implanted stem cell numbers and quality. Stem cell-mediated root regeneration hybridized with clinical crown technology may be a promising approach for functional tooth restoration owing to the availability of high-quality dental stem cells for autologous transplantation as well as long-term experience with clinical dental implant procedures. In addition, the orofacial region is an open system for directly delivering stem cells for tissue engineering. Our studies demonstrate that minipigs offer an excellent translational model for the concept of functional tooth regeneration and the feasibility of using autologous stem cells for transplantation.

## Materials and Methods

### Subjects and Cell Culture

Normal human impacted third molars (n = 18) were collected from sixteen adults (18–20 yr of age) at the Dental Clinic of the National Institute of Dental & Craniofacial Research (NIDCR) under approved guidelines set by NIH Office of Human Subjects Research and University of Southern California IRB. Root apical papilla was gently separated from the surface of the root, minced and digested in a solution of 3 mg/ml collagenase type I (Worthington Biochemicals Corp., Freehold, NJ) and 4 mg/ml dispase (Roche Diagnostic/Boehringer Mannheim Corp., Indianapolis, IN) for 30 minutes at 37°C. Single cell suspensions of SCAP were obtained by passing through a 70 µm strainer (Falcon, BD Labware, Franklin Lakes, NJ), seeded at 1×10^4^ into 10 cm culture dishes (Costar, Cambridge, MA), and cultured with alpha-Modification of Eagle's Medium (GIBCO/Invitrogen, Carlsbad, CA) supplemented with 15% FBS (Equitech-Bio Inc., Kerrville, TX), 100 µM L-ascorbic acid 2-phosphate (WAKO, Tokyo, Japan), 2 mM L-glutamine (Biosource/Invitrogen), 100 U/ml penicillin and 100 µg/ml streptomycin at 37°C in 5% CO_2_. To assess colony-forming efficiency, day 10 cultures were fixed with 4% formalin, and then stained with 0.1% toluidine blue. Aggregates of ≥50 cells were scored as colonies. The proliferation rate of sub-confluent cultures (first passage) of SCAP was assessed by BrdU incorporation for 6 hours, using BrdU staining Kit (Zymed/Invitrogen). Conditions for the induction of calcium accumulation were as reported previously [18]. Calcium accumulation was detected by 2% Alizarin Red S (pH 4.2) staining. The induction of adipogenesis was as previously reported [8]. DPSCs and PDLSCs were isolated and cultured as previously described [8], [9]. In some experiments, SCAP, DPSCs, and PDLSCs were obtained from the same donor or donors. All primary cells used in this study were at 1–3 passages. For each experiment, same passage of SCAP, DPSCs, and PDLSCs were used.

### Antibodies

Rabbit antibodies included anti-HSP90 (Santa Cruz Biotechnology, Inc., Santa Cruz, CA); anti-Cbfa1 (EMD Biosciences, Inc., San Diego, CA); anti-human-specific mitochondria (Chemicon, Temecula, CA). Mouse antibodies included anti-survivin (Santa Cruz Biotechnology, Inc.); anti-DSP (LF-21) (Dr. Larry Fisher, NIDCR/NIH); anti-CD146 and STRO-1 (Dr. Stan Gronthos, Institute of Medical and Veterinary Science, Australia). Rabbit and murine isotype-matched negative control antibodies were obtained from Caltag Laboratories (Caltag/Invitrogen). For flow cytometric analysis, R-PE conjugated monoclonal anti-human antibodies include: CD14, CD18, CD24, CD29, CD34, CD35, CD73, CD90, CD105, CD106, CD146, CD150, CD166 were purchased from Pharmingen/BD Bioscience (San Jose, CA) and monoclonal anti-human ALP was from Hybridoma Bank (Iowa University, Iowa).

### Transplantation

Approximately 4.0×10^6^ of *in vitro* expanded SCAP, DPSCs, and PDLSCs were mixed with 40 mg of HA/TCP ceramic particles (Zimmer Inc, Warsaw, IN) and then transplanted subcutaneously into the dorsal surface of 10-week-old immunocompromised beige mice (NIH-bg-nu/nu-xid, Harlan Sprague Dawley, Indianapolis, IN) as previously described [8], [9], [18], [19]. These procedures were performed in accordance with specifications of approved animal protocols (NIDCR #04-317 and USC #10874). The transplants were recovered after 8 weeks, fixed with 4% formalin, decalcified with buffered 10% EDTA (pH 8.0), and then embedded in paraffin. Sections were deparaffinized and stained with H&E.

### Immunohistochemistry

Deparaffinized sections were immersed in 3% H_2_O_2_/methanol for 15 minutes to quench the endogenous peroxidase activity, and incubated with primary antibodies (1∶200 to 1∶500 dilution). Isotype-matched control antibodies were used under the same conditions for 1 hour. For enzymatic immunohistochemical staining, Zymed SuperPicTure polymer detection kit (Zymed/Invitrogen) was used according to the manufacturer's protocol. Subsequently, sections were counterstained with hematoxylin.

### Telomerase Activity

Telomerase activity in SCAP, DPSCs and BMSSCs was detected by using a quantitative telomerase detection kit (Allied Biotech, Inc., Ijamsville, MD) according to the manufacture's protocol for real time PCR. Briefly, the telomerase in the cell extract from 1×10^5^ cells of SCAP, DPSCs, or BMSSCs added telomeric repeats (TTAGGG) onto the 3′ end of the substrate oligonucleotide and iQ SYBR Green Supermix (BioRad Laboratories, Hercules, CA), and amplified with an iCycler iQ real-time PCR machine (BioRad Laboratories). The generated PCR products are directly detected by measuring the increase in fluorescence caused by binding of SYBR Green to double-strand DNA and calculated by using an iCycler iQ software (BioRad Laboratories). Some cell extract was heated at 85°C for 10 minutes and used for negative control. The real-time PCR condition was as follows; telomerase reaction for 20 minutes at 25°C, PCR initial activation step for 3 minutes at 95°C, 3-step cycling; denaturation for 10 seconds at 95°C, annealing for 30 seconds at 60°C, extension for 3 minutes at 72°C, and cycle number 40 cycles.

### Population doubling

SCAP and DPSCs were cultured at low density to form single cell-derived colonies and then were trypsinized and seeded at a density of 0.5×10^4^ cells in 100-mm culture dishes at the first passage. Upon reaching confluency, the cells were trypsinized and seeded at the same cell density. The population doubling was calculated at every passage according to the equation: log_2_ (the number of harvested cells/the number of seeded cells). The finite population doublings were determined by cumulative addition of total numbers generated from each passage until the cells ceased dividing [20]. The criteria for cell senescence are that cells do not divide for a month in culture and that over 60% of the cells are stained positive for beta-galactosidase [18].

### Flow Cytometric Analysis

The procedure for flow cytometric analysis was performed as described previously [16]. Briefly, cells were trypsinized, and approximately 2×10^5^ cells were pelleted in 5-mL polypropylene tubes (BD Bioscience). They were treated with 5% heat-inactivated FBS, 1% BSA (ICN Biomedicals, Inc., Aurora, OH) and 5% normal human serum (Jackson Immuno Research Laboratories, Inc., West Grove, PA) in HBSS (GIBCO/Invitrogen) for 30 minutes on ice to block non-specific binding sites. For STRO-1 and ALP staining, some of the cells were incubated with 100 µl of STRO-1 supernatant (mouse IgM anti-human STRO-1) [16] or mouse IgG_1_ anti-human bone/liver/kidney ALP (Hybridoma Bank) for 45 minutes on ice. After washing with 5% heat-inactivated FBS in HBSS at 4°C, they were reacted with R-PE conjugated goat F(ab′)_2_ anti-mouse IgM (µ chain specific) (Biosource/Invitrogen) or anti-mouse IgG (H+L) (Southern Biotechnology Associates, Inc., Birmingham, AL) for 30 minutes on ice. For CD24 staining, the cells were treated with 1 µg of R-PE conjugated mouse IgG_1_ anti-mouse CD24 (BD Bioscience) for 45 minutes on ice. As negative controls, some of the cells were incubated with 1 µg of non-immune mouse IgM (Southern Biotechnology Associates, Inc.) or IgG_1_ (BD Bioscience). After washing, all of the cells were then sorted on a FACSCalibur flow cytometer (BD Bioscience) by collecting 10,000 events, and analyzed by means of a Cell Quest software (BD Bioscience).

### Swine Model

Six inbred male minipigs (4–8 month-old, weighing 20–40 kg) were obtained from the Institute of Animal Science of the Chinese Agriculture University. At this age, minipig incisors are still developing. Minipigs were kept under conventional conditions with free access to water and regularly supply of soft food. This study was reviewed and approved by the Animal Care and Use Committees of Capital University of Medical Sciences and the Institute of Dental and Craniofacial Research. Minipigs were anaesthetized with a combination of ketamine chloride (6 mg/kg) and xylazine (0.6 mg/kg) before the surgery. Minipig SCAP and PDLSCs were isolated and cultured the same as human SCAP and PDLSCs as mentioned above.

### Western Blotting

Primary antibodies were used at dilutions ranging from 1∶200 to 1∶1000. Western blot analyses were carried out as previously reported [18], [21].

### Compressive Strength Measurement

Compressive strength was tested by H5KS type force test system with loading of 1mm/min (Tinius Olsen H5KS testing machine, Tinius Olsen, Ltd., Survey, UK). A newly formed bio-root was harvested six months after transplant, and divided into three pieces. Compressive strength of each piece was measured separately. Compressive strength of natural minipig roots and original HA/TCP carriers were also measured (n = 5, each group).

### Statistical Analysis

Student *t*-test was used to analyze the differences between groups. One-way repeated ANOVA followed by Scheffe's comparison was used to compare data from three or more groups. *P* values less than 0.05 were considered as statistically significant. All statistical data are presented as mean±SD (n = 5).
